# 3,4,5-Trihydr­oxy-*N*′-(1*H*-indol-2-ylmethyl­idene)benzohydrazide–1*H*-indole-2-carbaldehyde azine–methanol (2/1/2)

**DOI:** 10.1107/S1600536809052465

**Published:** 2009-12-12

**Authors:** Hamid Khaledi, Abeer A. Alhadi, Hapipah Mohd Ali, Ward T. Robinson, Mahmood A. Abdulla

**Affiliations:** a Department of Chemistry, University of Malaya, 50603 Kuala Lumpur, Malaysia; b Department of Molecular Medicine, University of Malaya, 50603 Kuala Lumpur, Malaysia

## Abstract

The title compound, 2C_16_H_13_N_3_O_4_·C_18_H_14_N_4_·2CH_4_O, was crystallized from the reaction between 3,4,5-trihydroxy­benzoyl­hydrazine and indole-2-carbaldehyde in a mixture of ethanol and methanol. The compound is a stoichiometric 2:1 cocrystal of the methanol-solvated reaction product, 3,4,5-trihydr­oxy-*N*′-(1*H*-indol-2-ylmethyl­idene)benzohydrazide and 1*H*-indole-2-carbaldehyde azine that arose unexpectedly during the synthesis. The former mol­ecules are linked by O—H⋯O hydrogen bonds and also by π–π stacking inter­actions between benzoyl­hydrazide rings into a two-dimensional network. The methanol solvent mol­ecules are hydrogen bonded to this network. The centrosymmetric azine mol­ecules are not engaged in hydrogen bonding.

## Related literature

For the crystal structures of some compounds similar to 3,4,5-trihydr­oxy-*N′*-[(1*H*-indol-2-yl)methyl­idene]benzoyl­hydrazide, see: Khaledi *et al.* (2008*a* ▶,*b* ▶, 2009*a* ▶,*b* ▶). For the structure of 1*H*-indole-2-carbaldehyde azine, see: Rizal *et al.* (2008 ▶). For the biological activity of gallic acid (3,4,5-trihydroxybenzoic acid) derivatives see: Arunkumar *et al.* (2006 ▶); Saxena *et al.* (2008 ▶).
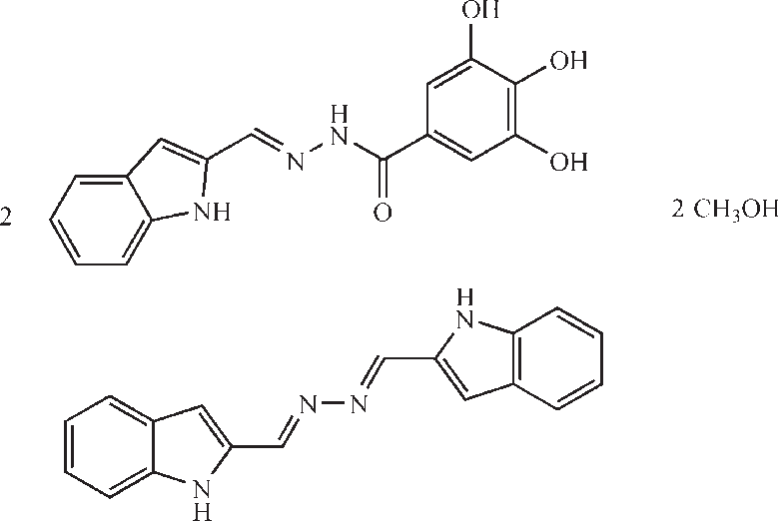


## Experimental

### 

#### Crystal data

2C_16_H_13_N_3_O_4_·C_18_H_14_N_4_·2CH_4_O
*M* *_r_* = 973.00
Triclinic, 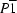

*a* = 7.4642 (15) Å
*b* = 12.791 (2) Å
*c* = 25.079 (5) Å
α = 95.918 (3)°
β = 95.166 (4)°
γ = 101.451 (4)°
*V* = 2319.3 (8) Å^3^
*Z* = 2
Mo *K*α radiation
μ = 0.10 mm^−1^
*T* = 100 K
0.50 × 0.12 × 0.03 mm

#### Data collection

Bruker APEXII CCD diffractometer
Absorption correction: multi-scan (*SADABS*; Sheldrick, 1996 ▶) *T*_min_ = 0.952, *T*_max_ = 0.997
10985 measured reflections
7939 independent reflections
4029 reflections with *I* > 2σ(*I*)
*R*_int_ = 0.054

#### Refinement

*R*[*F* ^2^ > 2σ(*F* ^2^)] = 0.064
*wR*(*F* ^2^) = 0.153
*S* = 0.98
7939 reflections
693 parameters
14 restraints
H atoms treated by a mixture of independent and constrained refinement
Δρ_max_ = 0.30 e Å^−3^
Δρ_min_ = −0.32 e Å^−3^

### 

Data collection: *APEX2* (Bruker, 2007 ▶); cell refinement: *SAINT* (Bruker, 2007 ▶); data reduction: *SAINT*; program(s) used to solve structure: *SHELXS97* (Sheldrick, 2008 ▶); program(s) used to refine structure: *SHELXL97* (Sheldrick, 2008 ▶); molecular graphics: *X-SEED* (Barbour, 2001 ▶) and *Mercury* (Macrae *et al.*, 2008 ▶); software used to prepare material for publication: *publCIF* (Westrip, 2009 ▶).

## Supplementary Material

Crystal structure: contains datablocks I, global. DOI: 10.1107/S1600536809052465/om2302sup1.cif

Structure factors: contains datablocks I. DOI: 10.1107/S1600536809052465/om2302Isup2.hkl

Additional supplementary materials:  crystallographic information; 3D view; checkCIF report

## Figures and Tables

**Table 1:** Hydrogen-bond geometry (Å, °)

*D*—H⋯*A*	*D*—H	H⋯*A*	*D*⋯*A*	*D*—H⋯*A*
O13—H13*O*⋯O14	0.84 (1)	2.21 (4)	2.674 (4)	115 (4)
O14—H14*O*⋯O31^i^	0.84 (1)	1.98 (2)	2.778 (4)	158 (4)
O14—H14*O*⋯O15	0.84 (1)	2.33 (4)	2.763 (4)	112 (3)
O15—H15*O*⋯O26^ii^	0.85 (1)	1.77 (1)	2.617 (4)	179 (4)
N1—H1*N*⋯O51^iii^	0.88 (1)	2.11 (1)	2.976 (4)	167 (3)
N3—H3*N*⋯O52^iv^	0.88 (1)	2.13 (2)	2.983 (4)	165 (4)
O29—H29*O*⋯O30^v^	0.85 (1)	2.11 (2)	2.858 (4)	148 (4)
O30—H30*O*⋯O15^i^	0.84 (1)	1.91 (2)	2.732 (4)	165 (4)
O31—H31*O*⋯O10	0.84 (1)	1.80 (1)	2.626 (4)	172 (4)
N4—H4*N*⋯O52^iii^	0.88 (1)	2.12 (1)	2.992 (4)	175 (3)
N6—H6*N*⋯O51^iii^	0.88 (1)	2.24 (2)	3.011 (4)	146 (3)
O51—H51*O*⋯O10^iii^	0.84 (1)	2.21 (3)	2.934 (4)	145 (4)
O51—H51*O*⋯N2^iii^	0.84 (1)	2.26 (3)	2.975 (4)	143 (4)
O52—H52*O*⋯N5^iii^	0.84 (1)	2.29 (3)	2.963 (4)	138 (4)
O52—H52*O*⋯O26^iii^	0.84 (1)	2.29 (2)	3.046 (4)	150 (4)

Symmetry codes: (i) 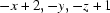; (ii) 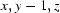; (iii) 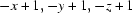; (iv) 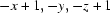; (v) 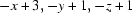.

**Fig. 1.**
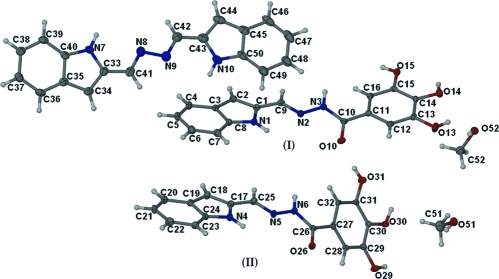
Perspective view of one crystal chemical unit of the title compound defining the atom labelling scheme and showing 40% probability displacement ellipsoids.

**Fig. 2.**
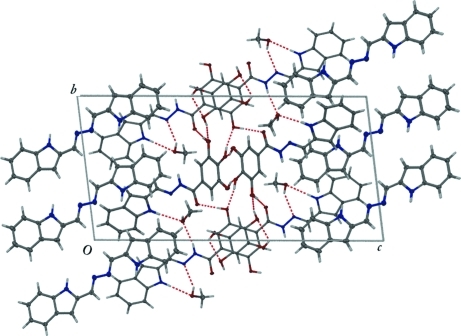
Packing view looking down the crystallographic α unit cell edge and showing all the links, between electronegative O and N atoms, which are within the range for normal hydrogen bonds.

**Fig. 3.**
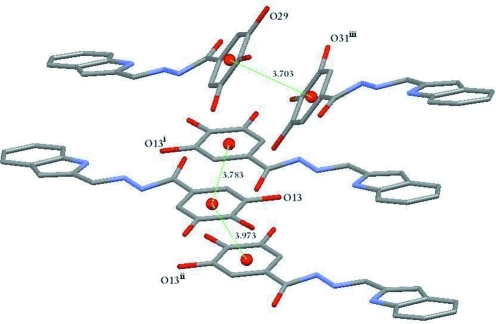
A view of the intermolecular π–π interactions between gallic hydrazone molecules. H atoms have been omitted for clarity. [Symmetry codes: (i) -x + 2, -y, -z + 1; (ii) -x + 1, -y, -z + 1; (iii) -x + 2, -y + 1, -z + 1.]

**Fig. 4.**
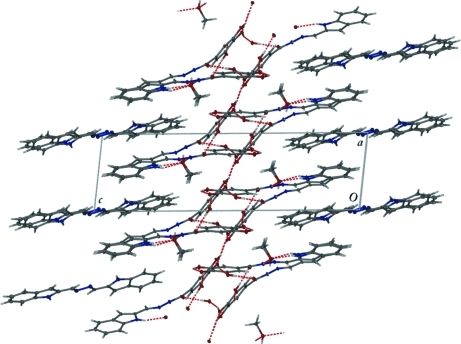
Packing view looking down the crystallographic b unit cell edge thus emphasizing the two-dimensional extension of the polymeric network and the interdigitation with hydrazine molecules.

## supplementary crystallographic information

### Comment

Gallic acid (3,4,5-trihydroxybenzoic acid) derivatives have been studied for various biological activities including anticancer (Saxena *et al.*, 2008), antioxidant and antimicrobial activity (Arunkumar *et al.*, 2006). In order to prepare a new derivative of gallic acid, the reaction between 3,4,5-trihydroxybenzoylhydrazine and indole-2-carboxaldehyde was carried out to synthesize the related gallichydrazone; 3,4,5-trihydroxy-*N'*-[(1*H*-indol-2-yl)methylidene]benzoylhydrazide. However, a crystal suitable for X-ray diffraction was unexpectedly obtained during the synthesis.

A view of the title structure is illustrated in Fig. 1. The asymmetric unit contains two molecules of the gallic hydrazone with different conformation. In one of them, (I), the aromatic rings are nearly coplanar [dihedral angle = 11.00 (0.16) °], similar to the related previously reported structures (Khaledi *et al.*,  2008*a*, 2008b, 2009a, 2009b), whereas in the other one, (II), they are highly twisted with respect to each other, the dihedral angle between the two ring planes being 52.43 (0.11) °. The crystal structure is an infinite two-dimensional, hydrogen bonded, network of gallic hydrazone molecules (Fig. 2) with methanol solvate molecules. In addition, π–π interactions between aromatic rings occur. The benzoylhydrazide rings of molecule (I) and the symmetry-related planes at (-*x+*2, -*y*, -*z+*1 and -*x+*1, -*y*, -*z* + 1) are arranged in an antiparallel manner above each other in an infinite one dimensional chain with centroid separations of 3.783 (2) Å and 3.973 (2) Å alternatively. The benzoylhydrazide ring of molecule (II) and the symmetry-related plane at (-*x* + 2, -*y* + 1, -*z* + 1) also interact with one another through π–π stacking, with a centroid-centroid distances of 3.703 (2) Å, leading to a dimer. The framework is interdigitated with solvate azine molecules (Fig. 4) in the ratio of one for every two hydrazone molecules. The azine constituent, which is an unexpected decomposition product of the reaction, is almost planar [maximum deviation 0.21 (1) A°] with a *trans *configuration about N—N single bond, similar to its indole-3-carbaldehyde analogue (Rizal *et al.*, 2008).

### Experimental

A solution of indole-2-carboxaldehyde (0.725 g, 5 mmol) in methanol (20 ml) was added to a solution of 3,4,5-trihydroxybenzoylhydrazine (0.92 g, 5 mmol) in ethanol (60 ml). Furthermore, 1 ml of acetic acid was added and the mixture was refluxed for 4 h. The solution was then cooled and filtered to remove the unreacted hydrazide.The filtrate was set aside at room temperature overnight and crystals of the title compound were collected.

### Refinement

C-bound hydrogen atoms were placed at calculated positions (C–H 0.95 Å), and were treated as riding on their parent carbon atoms, with *U*(H) set to 1.2*U*eq(C). The nitrogen- and oxygen-bound H atoms were located in a difference Fourier map, and were refined with distance restraints of N–H 0.88±0.01 and O–H 0.84±0.01 Å.

### Crystal data

**Table:**

2C_16_H_13_N_3_O_4_·C_18_H_14_N_4_·2CH_4_O	*Z* = 2
*M**_r_* = 973.00	*F*(000) = 1020
Triclinic, *P*1	*D*_x_ = 1.393 Mg m^−^^3^
Hall symbol: -P 1	Mo *K*α radiation, λ = 0.71073 Å
*a* = 7.4642 (15) Å	Cell parameters from 754 reflections
*b* = 12.791 (2) Å	θ = 2.5–20.7°
*c* = 25.079 (5) Å	µ = 0.10 mm^−^^1^
α = 95.918 (3)°	*T* = 100 K
β = 95.166 (4)°	Lath, yellow
γ = 101.451 (4)°	0.50 × 0.12 × 0.03 mm
*V* = 2319.3 (8) Å^3^	

### Data collection

**Table:**

Bruker APEXII CCD diffractometer	7939 independent reflections
Radiation source: fine-focus sealed tube	4029 reflections with *I* > 2σ(*I*)
graphite	*R*_int_ = 0.054
φ and ω scans	θ_max_ = 25.0°, θ_min_ = 1.6°
Absorption correction: multi-scan (*SADABS*; Sheldrick, 1996)	*h* = −7→8
*T*_min_ = 0.952, *T*_max_ = 0.997	*k* = −15→13
10985 measured reflections	*l* = −29→29

### Refinement

**Table:**

Refinement on *F*^2^	Primary atom site location: structure-invariant direct methods
Least-squares matrix: full	Secondary atom site location: difference Fourier map
*R*[*F*^2^ > 2σ(*F*^2^)] = 0.064	Hydrogen site location: inferred from neighbouring sites
*wR*(*F*^2^) = 0.153	H atoms treated by a mixture of independent and constrained refinement
*S* = 0.98	*w* = 1/[σ^2^(*F*_o_^2^) + (0.0463*P*)^2^] where *P* = (*F*_o_^2^ + 2*F*_c_^2^)/3
7939 reflections	(Δ/σ)_max_ = 0.021
693 parameters	Δρ_max_ = 0.30 e Å^−^^3^
14 restraints	Δρ_min_ = −0.31 e Å^−^^3^

### Special details

**Table:**

Geometry. All e.s.d.'s (except the e.s.d. in the dihedral angle between two l.s. planes) are estimated using the full covariance matrix. The cell e.s.d.'s are taken into account individually in the estimation of e.s.d.'s in distances, angles and torsion angles; correlations between e.s.d.'s in cell parameters are only used when they are defined by crystal symmetry. An approximate (isotropic) treatment of cell e.s.d.'s is used for estimating e.s.d.'s involving l.s. planes.
Refinement. Refinement of *F*^2^ against ALL reflections. The weighted *R*-factor *wR* and goodness of fit *S* are based on *F*^2^, conventional *R*-factors *R* are based on *F*, with *F* set to zero for negative *F*^2^. The threshold expression of *F*^2^ > σ(*F*^2^) is used only for calculating *R*-factors(gt) *etc*. and is not relevant to the choice of reflections for refinement. *R*-factors based on *F*^2^ are statistically about twice as large as those based on *F*, and *R*- factors based on ALL data will be even larger.

### Fractional atomic coordinates and isotropic or equivalent isotropic displacement parameters (Å^2^)

**Table:**

	*x*	*y*	*z*	*U*_iso_*/*U*_eq_	
O10	0.7343 (4)	0.2306 (2)	0.60742 (10)	0.0276 (7)	
O13	0.7280 (4)	0.0510 (2)	0.41802 (11)	0.0333 (8)	
H13O	0.749 (6)	0.003 (3)	0.3953 (14)	0.050*	
O14	0.7874 (4)	−0.1457 (2)	0.42855 (11)	0.0280 (7)	
H14O	0.823 (6)	−0.201 (2)	0.4370 (17)	0.042*	
O15	0.8285 (4)	−0.2137 (2)	0.52914 (10)	0.0259 (7)	
H15O	0.846 (6)	−0.225 (3)	0.5616 (6)	0.039*	
N1	0.5730 (5)	0.2917 (3)	0.78752 (12)	0.0211 (8)	
H1N	0.571 (5)	0.321 (3)	0.7570 (8)	0.025*	
N2	0.6671 (5)	0.1652 (2)	0.70038 (13)	0.0242 (8)	
N3	0.6927 (5)	0.0943 (3)	0.65781 (13)	0.0260 (8)	
H3N	0.668 (5)	0.0250 (10)	0.6599 (15)	0.031*	
C1	0.6341 (5)	0.1973 (3)	0.79275 (15)	0.0203 (9)	
C2	0.6431 (5)	0.1802 (3)	0.84590 (15)	0.0263 (10)	
H2	0.6815	0.1215	0.8604	0.032*	
C3	0.5848 (5)	0.2656 (3)	0.87524 (15)	0.0232 (10)	
C4	0.5625 (6)	0.2922 (3)	0.92965 (16)	0.0290 (11)	
H4	0.5937	0.2486	0.9560	0.035*	
C5	0.4951 (6)	0.3823 (3)	0.94423 (16)	0.0317 (11)	
H5	0.4791	0.4007	0.9809	0.038*	
C6	0.4495 (6)	0.4473 (3)	0.90547 (17)	0.0325 (11)	
H6	0.4025	0.5088	0.9167	0.039*	
C7	0.4705 (5)	0.4250 (3)	0.85215 (16)	0.0263 (10)	
H7	0.4386	0.4696	0.8263	0.032*	
C8	0.5402 (5)	0.3347 (3)	0.83727 (15)	0.0222 (10)	
C9	0.6730 (5)	0.1316 (3)	0.74681 (16)	0.0258 (10)	
H9	0.7028	0.0639	0.7509	0.031*	
C10	0.7225 (6)	0.1329 (3)	0.61037 (16)	0.0235 (10)	
C11	0.7412 (5)	0.0573 (3)	0.56412 (15)	0.0199 (9)	
C12	0.7246 (5)	0.0893 (3)	0.51285 (15)	0.0219 (10)	
H12	0.7020	0.1585	0.5083	0.026*	
C13	0.7412 (5)	0.0194 (3)	0.46881 (15)	0.0207 (9)	
C14	0.7731 (5)	−0.0820 (3)	0.47460 (15)	0.0203 (9)	
C15	0.7928 (5)	−0.1131 (3)	0.52580 (15)	0.0191 (9)	
C16	0.7771 (5)	−0.0445 (3)	0.57041 (15)	0.0223 (10)	
H16	0.7907	−0.0661	0.6054	0.027*	
O26	0.8842 (4)	0.7516 (2)	0.62971 (10)	0.0246 (7)	
O29	1.3673 (4)	0.6412 (2)	0.51863 (11)	0.0276 (7)	
H29O	1.430 (5)	0.601 (3)	0.5035 (15)	0.041*	
O30	1.3089 (4)	0.4224 (2)	0.51396 (10)	0.0225 (7)	
H30O	1.254 (5)	0.3578 (13)	0.5053 (15)	0.034*	
O31	1.0303 (4)	0.3140 (2)	0.56466 (11)	0.0279 (7)	
H31O	0.938 (4)	0.293 (3)	0.5802 (15)	0.042*	
N4	0.5999 (5)	0.7939 (3)	0.79555 (12)	0.0230 (8)	
H4N	0.609 (5)	0.820 (3)	0.7648 (8)	0.028*	
N5	0.7553 (5)	0.6765 (2)	0.71588 (12)	0.0243 (8)	
N6	0.8261 (5)	0.6142 (3)	0.67896 (13)	0.0240 (8)	
H6N	0.818 (5)	0.5464 (12)	0.6837 (15)	0.029*	
C17	0.6739 (5)	0.7044 (3)	0.80452 (15)	0.0233 (10)	
C18	0.6735 (5)	0.6888 (3)	0.85733 (15)	0.0241 (10)	
H18	0.7163	0.6331	0.8736	0.029*	
C19	0.5975 (5)	0.7705 (3)	0.88380 (15)	0.0232 (10)	
C20	0.5626 (6)	0.7976 (3)	0.93695 (16)	0.0284 (11)	
H20	0.5955	0.7576	0.9648	0.034*	
C21	0.4798 (6)	0.8830 (3)	0.94795 (16)	0.0312 (11)	
H21	0.4562	0.9024	0.9838	0.037*	
C22	0.4298 (6)	0.9418 (3)	0.90718 (17)	0.0315 (11)	
H22	0.3710	0.9996	0.9160	0.038*	
C23	0.4630 (5)	0.9184 (3)	0.85492 (16)	0.0250 (10)	
H23	0.4264	0.9583	0.8274	0.030*	
C24	0.5516 (5)	0.8349 (3)	0.84352 (15)	0.0209 (9)	
C25	0.7390 (5)	0.6424 (3)	0.76214 (15)	0.0233 (10)	
H25	0.7701	0.5758	0.7683	0.028*	
C26	0.8945 (5)	0.6580 (3)	0.63666 (15)	0.0209 (9)	
C27	0.9906 (5)	0.5921 (3)	0.60161 (14)	0.0201 (9)	
C28	1.1271 (5)	0.6445 (3)	0.57419 (14)	0.0198 (9)	
H28	1.1494	0.7206	0.5756	0.024*	
C29	1.2315 (5)	0.5871 (3)	0.54470 (15)	0.0209 (9)	
C30	1.1997 (5)	0.4760 (3)	0.54255 (14)	0.0183 (9)	
C31	1.0592 (5)	0.4240 (3)	0.56948 (14)	0.0185 (9)	
C32	0.9561 (5)	0.4800 (3)	0.59929 (14)	0.0188 (9)	
H32	0.8627	0.4434	0.6181	0.023*	
N7	−0.1199 (5)	0.3424 (3)	1.11800 (14)	0.0329 (9)	
H7N	−0.160 (5)	0.2730 (11)	1.1082 (15)	0.039*	
N8	−0.0309 (5)	0.2620 (3)	1.01631 (15)	0.0412 (10)	
N9	0.0213 (5)	0.2391 (3)	0.96486 (15)	0.0413 (10)	
N10	0.0984 (5)	0.1700 (3)	0.86232 (16)	0.0390 (10)	
H10N	0.140 (6)	0.2386 (12)	0.8734 (17)	0.047*	
C33	−0.0304 (6)	0.4065 (4)	1.08362 (17)	0.0338 (11)	
C34	0.0234 (6)	0.5092 (4)	1.10970 (17)	0.0338 (11)	
H34	0.0883	0.5694	1.0952	0.041*	
C35	−0.0343 (6)	0.5100 (4)	1.16199 (17)	0.0318 (11)	
C36	−0.0175 (6)	0.5877 (4)	1.20641 (18)	0.0365 (12)	
H36	0.0427	0.6598	1.2047	0.044*	
C37	−0.0892 (6)	0.5589 (4)	1.25316 (18)	0.0364 (12)	
H37	−0.0783	0.6116	1.2835	0.044*	
C38	−0.1782 (6)	0.4520 (4)	1.25592 (18)	0.0355 (12)	
H38	−0.2267	0.4337	1.2883	0.043*	
C39	−0.1965 (6)	0.3734 (4)	1.21283 (17)	0.0342 (11)	
H39	−0.2565	0.3014	1.2151	0.041*	
C40	−0.1247 (6)	0.4023 (3)	1.16585 (17)	0.0299 (11)	
C41	0.0086 (6)	0.3637 (4)	1.03193 (17)	0.0351 (12)	
H41	0.0648	0.4113	1.0087	0.042*	
C42	−0.0074 (6)	0.1373 (4)	0.94922 (19)	0.0404 (12)	
H42	−0.0528	0.0874	0.9729	0.048*	
C43	0.0283 (6)	0.0996 (4)	0.8971 (2)	0.0404 (12)	
C44	−0.0064 (6)	−0.0023 (4)	0.8699 (2)	0.0450 (13)	
H44	−0.0547	−0.0667	0.8842	0.054*	
C45	0.0423 (6)	0.0051 (4)	0.8166 (2)	0.0388 (12)	
C46	0.0367 (6)	−0.0702 (4)	0.7703 (2)	0.0505 (15)	
H46	−0.0038	−0.1450	0.7716	0.061*	
C47	0.0905 (7)	−0.0332 (5)	0.7238 (2)	0.0575 (16)	
H47	0.0874	−0.0831	0.6927	0.069*	
C48	0.1500 (6)	0.0768 (4)	0.7210 (2)	0.0519 (15)	
H48	0.1842	0.1002	0.6879	0.062*	
C49	0.1598 (6)	0.1511 (4)	0.76502 (19)	0.0454 (13)	
H49	0.2018	0.2256	0.7631	0.055*	
C50	0.1067 (6)	0.1146 (4)	0.81268 (19)	0.0379 (12)	
O51	0.3727 (4)	0.6124 (2)	0.31454 (11)	0.0291 (7)	
H51O	0.348 (6)	0.6718 (18)	0.3249 (16)	0.044*	
C51	0.5272 (6)	0.6038 (4)	0.35040 (18)	0.0448 (13)	
H51A	0.6326	0.6607	0.3464	0.067*	
H51B	0.5580	0.5335	0.3418	0.067*	
H51C	0.4971	0.6114	0.3876	0.067*	
O52	0.3718 (4)	0.1291 (2)	0.31264 (11)	0.0270 (7)	
H52O	0.303 (5)	0.173 (3)	0.3177 (17)	0.040*	
C52	0.5328 (6)	0.1969 (3)	0.34338 (17)	0.0348 (11)	
H52A	0.5520	0.2685	0.3315	0.052*	
H52B	0.6404	0.1656	0.3378	0.052*	
H52C	0.5157	0.2028	0.3818	0.052*	

### Atomic displacement parameters (Å^2^)

**Table:**

	*U*^11^	*U*^22^	*U*^33^	*U*^12^	*U*^13^	*U*^23^
O10	0.0363 (18)	0.0170 (17)	0.0324 (17)	0.0056 (13)	0.0150 (14)	0.0060 (13)
O13	0.052 (2)	0.034 (2)	0.0181 (17)	0.0156 (16)	0.0047 (15)	0.0088 (13)
O14	0.0370 (19)	0.0241 (18)	0.0239 (16)	0.0105 (14)	0.0041 (14)	−0.0008 (13)
O15	0.0401 (19)	0.0190 (16)	0.0207 (16)	0.0095 (14)	0.0071 (15)	0.0033 (13)
N1	0.025 (2)	0.023 (2)	0.0172 (19)	0.0072 (16)	0.0037 (16)	0.0043 (15)
N2	0.034 (2)	0.0181 (19)	0.021 (2)	0.0060 (16)	0.0088 (16)	0.0013 (15)
N3	0.036 (2)	0.0176 (19)	0.024 (2)	0.0029 (17)	0.0120 (17)	−0.0006 (16)
C1	0.019 (2)	0.019 (2)	0.025 (2)	0.0060 (18)	0.0048 (18)	0.0023 (18)
C2	0.029 (3)	0.024 (3)	0.024 (2)	0.003 (2)	0.001 (2)	0.0049 (19)
C3	0.028 (3)	0.019 (2)	0.021 (2)	0.0014 (19)	0.0034 (19)	0.0030 (18)
C4	0.036 (3)	0.031 (3)	0.018 (2)	0.004 (2)	0.003 (2)	0.0018 (19)
C5	0.041 (3)	0.033 (3)	0.019 (2)	0.004 (2)	0.005 (2)	−0.003 (2)
C6	0.032 (3)	0.033 (3)	0.031 (3)	0.004 (2)	0.008 (2)	−0.005 (2)
C7	0.025 (3)	0.025 (3)	0.029 (3)	0.007 (2)	0.003 (2)	0.0004 (19)
C8	0.022 (2)	0.024 (2)	0.019 (2)	0.0013 (19)	0.0024 (18)	0.0006 (18)
C9	0.026 (3)	0.024 (3)	0.026 (2)	0.0009 (19)	0.004 (2)	0.0033 (19)
C10	0.025 (2)	0.017 (2)	0.027 (2)	−0.0002 (19)	0.0068 (19)	0.0010 (18)
C11	0.019 (2)	0.018 (2)	0.023 (2)	0.0029 (18)	0.0064 (18)	0.0027 (18)
C12	0.026 (2)	0.015 (2)	0.025 (2)	0.0039 (18)	0.0035 (19)	0.0062 (18)
C13	0.021 (2)	0.025 (2)	0.017 (2)	0.0053 (19)	0.0022 (18)	0.0061 (18)
C14	0.020 (2)	0.022 (2)	0.016 (2)	0.0013 (18)	0.0054 (18)	−0.0020 (18)
C15	0.021 (2)	0.012 (2)	0.025 (2)	0.0049 (17)	0.0039 (18)	0.0047 (17)
C16	0.026 (2)	0.019 (2)	0.022 (2)	0.0021 (19)	0.0061 (19)	0.0043 (18)
O26	0.0384 (18)	0.0154 (16)	0.0228 (15)	0.0097 (13)	0.0091 (13)	0.0030 (12)
O29	0.0279 (18)	0.0197 (17)	0.0383 (18)	0.0050 (13)	0.0181 (14)	0.0051 (13)
O30	0.0223 (17)	0.0174 (16)	0.0282 (16)	0.0030 (13)	0.0085 (13)	0.0017 (13)
O31	0.0291 (18)	0.0205 (17)	0.0366 (18)	0.0061 (14)	0.0190 (14)	0.0000 (13)
N4	0.026 (2)	0.025 (2)	0.019 (2)	0.0074 (16)	0.0045 (17)	0.0001 (16)
N5	0.032 (2)	0.025 (2)	0.0176 (19)	0.0059 (16)	0.0091 (16)	0.0011 (15)
N6	0.035 (2)	0.019 (2)	0.0201 (19)	0.0070 (17)	0.0120 (16)	0.0019 (16)
C17	0.021 (2)	0.026 (3)	0.024 (2)	0.0051 (19)	0.0069 (19)	0.0025 (19)
C18	0.028 (3)	0.027 (2)	0.021 (2)	0.011 (2)	0.0047 (19)	0.0057 (18)
C19	0.026 (2)	0.022 (2)	0.022 (2)	0.0047 (19)	0.0036 (19)	0.0035 (18)
C20	0.034 (3)	0.029 (3)	0.021 (2)	0.004 (2)	0.004 (2)	0.0020 (19)
C21	0.041 (3)	0.031 (3)	0.020 (2)	0.005 (2)	0.010 (2)	−0.004 (2)
C22	0.032 (3)	0.027 (3)	0.038 (3)	0.011 (2)	0.010 (2)	0.001 (2)
C23	0.030 (3)	0.020 (2)	0.028 (2)	0.010 (2)	0.008 (2)	0.0051 (19)
C24	0.021 (2)	0.022 (2)	0.017 (2)	0.0028 (19)	0.0016 (18)	−0.0025 (18)
C25	0.024 (2)	0.023 (2)	0.025 (2)	0.0063 (19)	0.0065 (19)	0.0055 (19)
C26	0.022 (2)	0.018 (2)	0.021 (2)	0.0015 (18)	0.0012 (18)	0.0011 (18)
C27	0.020 (2)	0.025 (2)	0.014 (2)	0.0049 (19)	0.0011 (17)	−0.0009 (17)
C28	0.030 (2)	0.010 (2)	0.020 (2)	0.0056 (18)	0.0037 (19)	0.0026 (17)
C29	0.019 (2)	0.019 (2)	0.024 (2)	0.0018 (18)	0.0028 (19)	0.0010 (18)
C30	0.014 (2)	0.022 (2)	0.018 (2)	0.0046 (18)	0.0054 (17)	−0.0017 (17)
C31	0.021 (2)	0.012 (2)	0.021 (2)	0.0011 (18)	0.0031 (18)	0.0022 (17)
C32	0.022 (2)	0.018 (2)	0.017 (2)	0.0022 (18)	0.0083 (18)	0.0044 (17)
N7	0.030 (2)	0.029 (2)	0.037 (2)	−0.0003 (19)	0.0046 (18)	0.0002 (19)
N8	0.036 (2)	0.050 (3)	0.035 (2)	0.005 (2)	0.0053 (19)	−0.001 (2)
N9	0.033 (2)	0.052 (3)	0.039 (2)	0.009 (2)	0.0062 (19)	0.003 (2)
N10	0.029 (2)	0.037 (3)	0.047 (3)	0.003 (2)	0.0061 (19)	−0.011 (2)
C33	0.023 (3)	0.046 (3)	0.032 (3)	0.005 (2)	0.003 (2)	0.006 (2)
C34	0.026 (3)	0.035 (3)	0.042 (3)	0.006 (2)	−0.001 (2)	0.011 (2)
C35	0.021 (3)	0.038 (3)	0.037 (3)	0.008 (2)	−0.002 (2)	0.010 (2)
C36	0.030 (3)	0.028 (3)	0.048 (3)	0.000 (2)	−0.004 (2)	0.004 (2)
C37	0.033 (3)	0.039 (3)	0.035 (3)	0.008 (2)	−0.003 (2)	−0.001 (2)
C38	0.023 (3)	0.044 (3)	0.037 (3)	0.002 (2)	0.000 (2)	0.009 (2)
C39	0.024 (3)	0.032 (3)	0.042 (3)	−0.001 (2)	−0.003 (2)	0.005 (2)
C40	0.019 (2)	0.034 (3)	0.034 (3)	0.003 (2)	−0.002 (2)	0.001 (2)
C41	0.019 (3)	0.054 (3)	0.034 (3)	0.010 (2)	0.003 (2)	0.009 (2)
C42	0.030 (3)	0.047 (3)	0.045 (3)	0.009 (2)	−0.001 (2)	0.010 (3)
C43	0.031 (3)	0.038 (3)	0.052 (3)	0.008 (2)	0.004 (3)	0.003 (3)
C44	0.029 (3)	0.031 (3)	0.072 (4)	0.006 (2)	0.000 (3)	0.003 (3)
C45	0.022 (3)	0.035 (3)	0.056 (3)	0.007 (2)	−0.002 (2)	−0.006 (2)
C46	0.030 (3)	0.039 (3)	0.075 (4)	0.008 (2)	−0.008 (3)	−0.019 (3)
C47	0.037 (3)	0.063 (4)	0.064 (4)	0.009 (3)	0.007 (3)	−0.032 (3)
C48	0.038 (3)	0.053 (4)	0.057 (4)	−0.002 (3)	0.018 (3)	−0.020 (3)
C49	0.033 (3)	0.047 (3)	0.050 (3)	0.001 (2)	0.009 (3)	−0.012 (3)
C50	0.022 (3)	0.035 (3)	0.051 (3)	0.005 (2)	0.003 (2)	−0.016 (2)
O51	0.0360 (19)	0.0255 (18)	0.0272 (17)	0.0103 (15)	0.0070 (14)	−0.0004 (13)
C51	0.040 (3)	0.053 (3)	0.042 (3)	0.013 (3)	−0.004 (2)	0.005 (2)
O52	0.0322 (19)	0.0208 (18)	0.0285 (17)	0.0073 (13)	0.0044 (14)	0.0019 (13)
C52	0.027 (3)	0.040 (3)	0.034 (3)	0.002 (2)	−0.003 (2)	0.007 (2)

### Geometric parameters (Å, °)

**Table:**

O10—C10	1.246 (4)	C22—H22	0.9500
O13—C13	1.376 (4)	C23—C24	1.383 (5)
O13—H13O	0.844 (10)	C23—H23	0.9500
O14—C14	1.368 (4)	C25—H25	0.9500
O14—H14O	0.843 (10)	C26—C27	1.482 (5)
O15—C15	1.374 (4)	C27—C28	1.382 (5)
O15—H15O	0.845 (10)	C27—C32	1.400 (5)
N1—C8	1.373 (5)	C28—C29	1.383 (5)
N1—C1	1.387 (5)	C28—H28	0.9500
N1—H1N	0.884 (10)	C29—C30	1.388 (5)
N2—C9	1.282 (5)	C30—C31	1.393 (5)
N2—N3	1.380 (4)	C31—C32	1.370 (5)
N3—C10	1.354 (5)	C32—H32	0.9500
N3—H3N	0.877 (10)	N7—C40	1.363 (5)
C1—C2	1.371 (5)	N7—C33	1.376 (5)
C1—C9	1.440 (5)	N7—H7N	0.879 (10)
C2—C3	1.415 (5)	N8—C41	1.286 (5)
C2—H2	0.9500	N8—N9	1.400 (5)
C3—C4	1.405 (5)	N9—C42	1.290 (5)
C3—C8	1.426 (5)	N10—C43	1.378 (6)
C4—C5	1.375 (5)	N10—C50	1.380 (5)
C4—H4	0.9500	N10—H10N	0.877 (10)
C5—C6	1.404 (6)	C33—C34	1.371 (6)
C5—H5	0.9500	C33—C41	1.432 (6)
C6—C7	1.368 (5)	C34—C35	1.416 (6)
C6—H6	0.9500	C34—H34	0.9500
C7—C8	1.389 (5)	C35—C36	1.394 (6)
C7—H7	0.9500	C35—C40	1.425 (6)
C9—H9	0.9500	C36—C37	1.386 (6)
C10—C11	1.467 (5)	C36—H36	0.9500
C11—C12	1.392 (5)	C37—C38	1.409 (6)
C11—C16	1.401 (5)	C37—H37	0.9500
C12—C13	1.378 (5)	C38—C39	1.375 (6)
C12—H12	0.9500	C38—H38	0.9500
C13—C14	1.384 (5)	C39—C40	1.392 (6)
C14—C15	1.387 (5)	C39—H39	0.9500
C15—C16	1.377 (5)	C41—H41	0.9500
C16—H16	0.9500	C42—C43	1.414 (6)
O26—C26	1.243 (4)	C42—H42	0.9500
O29—C29	1.367 (4)	C43—C44	1.371 (6)
O29—H29O	0.845 (10)	C44—C45	1.425 (6)
O30—C30	1.369 (4)	C44—H44	0.9500
O30—H30O	0.841 (10)	C45—C50	1.405 (6)
O31—C31	1.372 (4)	C45—C46	1.423 (6)
O31—H31O	0.836 (10)	C46—C47	1.367 (7)
N4—C24	1.370 (5)	C46—H46	0.9500
N4—C17	1.396 (5)	C47—C48	1.399 (7)
N4—H4N	0.876 (10)	C47—H47	0.9500
N5—C25	1.289 (5)	C48—C49	1.367 (6)
N5—N6	1.369 (4)	C48—H48	0.9500
N6—C26	1.344 (5)	C49—C50	1.389 (6)
N6—H6N	0.877 (10)	C49—H49	0.9500
C17—C18	1.360 (5)	O51—C51	1.427 (5)
C17—C25	1.438 (5)	O51—H51O	0.839 (10)
C18—C19	1.419 (5)	C51—H51A	0.9800
C18—H18	0.9500	C51—H51B	0.9800
C19—C20	1.403 (5)	C51—H51C	0.9800
C19—C24	1.428 (5)	O52—C52	1.440 (5)
C20—C21	1.374 (5)	O52—H52O	0.837 (10)
C20—H20	0.9500	C52—H52A	0.9800
C21—C22	1.397 (6)	C52—H52B	0.9800
C21—H21	0.9500	C52—H52C	0.9800
C22—C23	1.369 (5)		
			
C13—O13—H13O	110 (3)	O26—C26—C27	122.7 (4)
C14—O14—H14O	109 (3)	N6—C26—C27	116.9 (4)
C15—O15—H15O	111 (3)	C28—C27—C32	120.0 (3)
C8—N1—C1	109.0 (3)	C28—C27—C26	118.3 (4)
C8—N1—H1N	128 (2)	C32—C27—C26	121.5 (3)
C1—N1—H1N	123 (2)	C27—C28—C29	120.5 (4)
C9—N2—N3	116.0 (3)	C27—C28—H28	119.7
C10—N3—N2	117.8 (3)	C29—C28—H28	119.7
C10—N3—H3N	121 (3)	O29—C29—C28	119.2 (3)
N2—N3—H3N	120 (3)	O29—C29—C30	120.8 (3)
C2—C1—N1	109.0 (3)	C28—C29—C30	120.0 (4)
C2—C1—C9	129.2 (4)	O30—C30—C29	118.2 (3)
N1—C1—C9	121.8 (4)	O30—C30—C31	122.9 (3)
C1—C2—C3	107.8 (4)	C29—C30—C31	118.9 (3)
C1—C2—H2	126.1	C32—C31—O31	121.9 (3)
C3—C2—H2	126.1	C32—C31—C30	121.7 (4)
C4—C3—C2	134.7 (4)	O31—C31—C30	116.4 (3)
C4—C3—C8	118.5 (4)	C31—C32—C27	118.9 (3)
C2—C3—C8	106.8 (3)	C31—C32—H32	120.5
C5—C4—C3	119.1 (4)	C27—C32—H32	120.5
C5—C4—H4	120.4	C40—N7—C33	109.8 (4)
C3—C4—H4	120.4	C40—N7—H7N	130 (3)
C4—C5—C6	120.7 (4)	C33—N7—H7N	120 (3)
C4—C5—H5	119.6	C41—N8—N9	111.2 (4)
C6—C5—H5	119.6	C42—N9—N8	112.7 (4)
C7—C6—C5	122.2 (4)	C43—N10—C50	110.2 (4)
C7—C6—H6	118.9	C43—N10—H10N	121 (3)
C5—C6—H6	118.9	C50—N10—H10N	128 (3)
C6—C7—C8	117.3 (4)	C34—C33—N7	108.4 (4)
C6—C7—H7	121.3	C34—C33—C41	129.2 (4)
C8—C7—H7	121.3	N7—C33—C41	122.2 (4)
N1—C8—C7	130.5 (4)	C33—C34—C35	108.3 (4)
N1—C8—C3	107.4 (3)	C33—C34—H34	125.9
C7—C8—C3	122.1 (4)	C35—C34—H34	125.9
N2—C9—C1	119.6 (4)	C36—C35—C34	135.0 (4)
N2—C9—H9	120.2	C36—C35—C40	119.0 (4)
C1—C9—H9	120.2	C34—C35—C40	106.0 (4)
O10—C10—N3	119.5 (3)	C37—C36—C35	119.5 (4)
O10—C10—C11	122.3 (4)	C37—C36—H36	120.3
N3—C10—C11	118.2 (4)	C35—C36—H36	120.3
C12—C11—C16	119.8 (3)	C36—C37—C38	120.4 (4)
C12—C11—C10	118.2 (4)	C36—C37—H37	119.8
C16—C11—C10	121.9 (4)	C38—C37—H37	119.8
C13—C12—C11	119.2 (4)	C39—C38—C37	121.5 (4)
C13—C12—H12	120.4	C39—C38—H38	119.3
C11—C12—H12	120.4	C37—C38—H38	119.3
O13—C13—C12	119.7 (3)	C38—C39—C40	118.1 (4)
O13—C13—C14	119.0 (3)	C38—C39—H39	120.9
C12—C13—C14	121.3 (4)	C40—C39—H39	120.9
O14—C14—C13	117.1 (3)	N7—C40—C39	131.0 (4)
O14—C14—C15	123.5 (4)	N7—C40—C35	107.5 (4)
C13—C14—C15	119.4 (3)	C39—C40—C35	121.5 (4)
O15—C15—C16	122.9 (4)	N8—C41—C33	121.3 (4)
O15—C15—C14	116.8 (3)	N8—C41—H41	119.3
C16—C15—C14	120.3 (4)	C33—C41—H41	119.3
C15—C16—C11	119.9 (4)	N9—C42—C43	120.3 (5)
C15—C16—H16	120.0	N9—C42—H42	119.8
C11—C16—H16	120.0	C43—C42—H42	119.8
C29—O29—H29O	113 (3)	C44—C43—N10	107.8 (4)
C30—O30—H30O	109 (3)	C44—C43—C42	130.9 (5)
C31—O31—H31O	107 (3)	N10—C43—C42	121.2 (4)
C24—N4—C17	108.0 (3)	C43—C44—C45	108.2 (4)
C24—N4—H4N	131 (3)	C43—C44—H44	125.9
C17—N4—H4N	120 (3)	C45—C44—H44	125.9
C25—N5—N6	116.1 (3)	C50—C45—C46	118.1 (5)
C26—N6—N5	118.8 (3)	C50—C45—C44	107.0 (4)
C26—N6—H6N	124 (3)	C46—C45—C44	134.9 (5)
N5—N6—H6N	117 (3)	C47—C46—C45	119.0 (5)
C18—C17—N4	109.7 (3)	C47—C46—H46	120.5
C18—C17—C25	128.0 (4)	C45—C46—H46	120.5
N4—C17—C25	122.2 (4)	C46—C47—C48	121.2 (5)
C17—C18—C19	107.8 (4)	C46—C47—H47	119.4
C17—C18—H18	126.1	C48—C47—H47	119.4
C19—C18—H18	126.1	C49—C48—C47	121.3 (5)
C20—C19—C18	134.9 (4)	C49—C48—H48	119.4
C20—C19—C24	118.8 (4)	C47—C48—H48	119.4
C18—C19—C24	106.3 (3)	C48—C49—C50	118.1 (5)
C21—C20—C19	118.8 (4)	C48—C49—H49	121.0
C21—C20—H20	120.6	C50—C49—H49	121.0
C19—C20—H20	120.6	N10—C50—C49	130.9 (4)
C20—C21—C22	121.0 (4)	N10—C50—C45	106.9 (4)
C20—C21—H21	119.5	C49—C50—C45	122.2 (4)
C22—C21—H21	119.5	C51—O51—H51O	106 (3)
C23—C22—C21	122.0 (4)	O51—C51—H51A	109.5
C23—C22—H22	119.0	O51—C51—H51B	109.5
C21—C22—H22	119.0	H51A—C51—H51B	109.5
C22—C23—C24	117.7 (4)	O51—C51—H51C	109.5
C22—C23—H23	121.2	H51A—C51—H51C	109.5
C24—C23—H23	121.2	H51B—C51—H51C	109.5
N4—C24—C23	130.2 (4)	C52—O52—H52O	95 (3)
N4—C24—C19	108.1 (3)	O52—C52—H52A	109.5
C23—C24—C19	121.6 (4)	O52—C52—H52B	109.5
N5—C25—C17	120.5 (4)	H52A—C52—H52B	109.5
N5—C25—H25	119.8	O52—C52—H52C	109.5
C17—C25—H25	119.8	H52A—C52—H52C	109.5
O26—C26—N6	120.4 (3)	H52B—C52—H52C	109.5

### Hydrogen-bond geometry (Å, °)

**Table:**

*D*—H···*A*	*D*—H	H···*A*	*D*···*A*	*D*—H···*A*
O13—H13O···O14	0.84 (1)	2.21 (4)	2.674 (4)	115 (4)
O14—H14O···O31^i^	0.84 (1)	1.98 (2)	2.778 (4)	158 (4)
O14—H14O···O15	0.84 (1)	2.33 (4)	2.763 (4)	112 (3)
O15—H15O···O26^ii^	0.85 (1)	1.77 (1)	2.617 (4)	179 (4)
N1—H1N···O51^iii^	0.88 (1)	2.11 (1)	2.976 (4)	167 (3)
N3—H3N···O52^iv^	0.88 (1)	2.13 (2)	2.983 (4)	165 (4)
O29—H29O···O30^v^	0.85 (1)	2.11 (2)	2.858 (4)	148 (4)
O30—H30O···O15^i^	0.84 (1)	1.91 (2)	2.732 (4)	165 (4)
O31—H31O···O10	0.84 (1)	1.80 (1)	2.626 (4)	172 (4)
N4—H4N···O52^iii^	0.88 (1)	2.12 (1)	2.992 (4)	175 (3)
N6—H6N···O51^iii^	0.88 (1)	2.24 (2)	3.011 (4)	146 (3)
O51—H51O···O10^iii^	0.84 (1)	2.21 (3)	2.934 (4)	145 (4)
O51—H51O···N2^iii^	0.84 (1)	2.26 (3)	2.975 (4)	143 (4)
O52—H52O···N5^iii^	0.84 (1)	2.29 (3)	2.963 (4)	138 (4)
O52—H52O···O26^iii^	0.84 (1)	2.29 (2)	3.046 (4)	150 (4)

Symmetry codes: (i) −*x*+2, −*y*, −*z*+1; (ii) *x*, *y*−1, *z*; (iii) −*x*+1, −*y*+1, −*z*+1; (iv) −*x*+1, −*y*, −*z*+1; (v) −*x*+3, −*y*+1, −*z*+1.
